# 2-(5-Bromo-3-isopropyl­sulfanyl-1-benzofuran-2-yl)acetic acid

**DOI:** 10.1107/S1600536811052160

**Published:** 2011-12-10

**Authors:** Pil Ja Seo, Hong Dae Choi, Byeng Wha Son, Uk Lee

**Affiliations:** a Department of Chemistry, Dongeui University, San 24 Kaya-dong Busanjin-gu, Busan 614-714, Republic of Korea; b Department of Chemistry, Pukyong National University, 599-1 Daeyeon 3-dong, Nam-gu, Busan 608-737, Republic of Korea

## Abstract

The title compound, C_13_H_13_BrO_3_S, was prepared by alkaline hydrolysis of ethyl 2-(5-bromo-3-isopropyl­sulfanyl-1-benzofuran-2-yl)acetate. In the crystal, the carboxyl groups are involved in inter­molecular O—H⋯O hydrogen bonds, which link the mol­ecules into dimers. These dimers are further packed into stacks along the *c* axis by inter­molecular C—H⋯π inter­actions, and by slipped π–π inter­actions between the furan rings of adjacent mol­ecules [centroid–centroid distance = 3.472 (2) Å, inter­planar distance = 3.398 (2) Å and slippage = 0.713 (2) Å].

## Related literature

For the pharmacological activity of benzofuran compounds, see: Aslam *et al.* (2009 ▶); Galal *et al.* (2009 ▶); Khan *et al.* (2005 ▶). For natural products with benzofuran rings, see: Akgul & Anil (2003 ▶); Soekamto *et al.* (2003 ▶). For the crystal structures of related compounds, see: Choi *et al.* (2009**a* ▶,b* ▶).
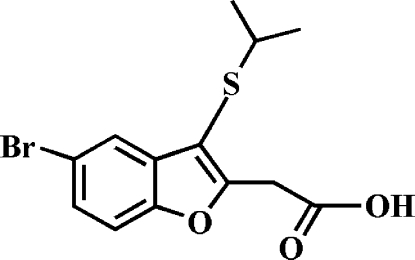


## Experimental

### 

#### Crystal data

C_13_H_13_BrO_3_S
*M* *_r_* = 329.20
Triclinic, 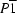

*a* = 7.4689 (3) Å
*b* = 9.9449 (4) Å
*c* = 10.0653 (4) Å
α = 98.415 (2)°
β = 102.146 (2)°
γ = 110.341 (3)°
*V* = 665.38 (5) Å^3^
*Z* = 2
Mo *K*α radiation
μ = 3.24 mm^−1^
*T* = 296 K
0.24 × 0.17 × 0.10 mm

#### Data collection

Bruker SMART APEXII CCD diffractometer
Absorption correction: multi-scan (*SADABS*; Bruker, 2009 ▶) *T*_min_ = 0.510, *T*_max_ = 0.738
11869 measured reflections
3082 independent reflections
2731 reflections with *I* > 2σ(*I*)
*R*_int_ = 0.030

#### Refinement

*R*[*F* ^2^ > 2σ(*F* ^2^)] = 0.024
*wR*(*F* ^2^) = 0.064
*S* = 1.03
3082 reflections
169 parameters
H atoms treated by a mixture of independent and constrained refinement
Δρ_max_ = 0.52 e Å^−3^
Δρ_min_ = −0.30 e Å^−3^

### 

Data collection: *APEX2* (Bruker, 2009 ▶); cell refinement: *SAINT* (Bruker, 2009 ▶); data reduction: *SAINT*; program(s) used to solve structure: *SHELXS97* (Sheldrick, 2008 ▶); program(s) used to refine structure: *SHELXL97* (Sheldrick, 2008 ▶); molecular graphics: *ORTEP-3* (Farrugia, 1997 ▶) and *DIAMOND* (Brandenburg, 1998 ▶); software used to prepare material for publication: *SHELXL97*.

## Supplementary Material

Crystal structure: contains datablock(s) global, I. DOI: 10.1107/S1600536811052160/bg2430sup1.cif

Structure factors: contains datablock(s) I. DOI: 10.1107/S1600536811052160/bg2430Isup2.hkl

Supplementary material file. DOI: 10.1107/S1600536811052160/bg2430Isup3.cml

Additional supplementary materials:  crystallographic information; 3D view; checkCIF report

## Figures and Tables

**Table 1:** Hydrogen-bond geometry (Å, °) *Cg*2 is the centroid of the C2–C7 ring.

*D*—H⋯*A*	*D*—H	H⋯*A*	*D*⋯*A*	*D*—H⋯*A*
O2—H2*O*⋯O3^i^	0.74 (3)	1.90 (3)	2.640 (2)	177 (3)
C9—H9*B*⋯*Cg*2^ii^	0.97	2.72 (1)	3.376 (2)	126

Symmetry codes: (i) 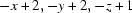; (ii) 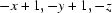.

**Fig. 1.**
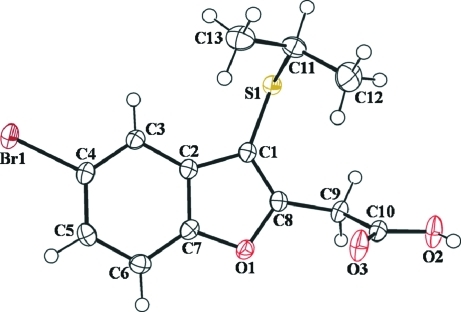
The molecular structure of the title compound with the atom numbering scheme. Displacement ellipsoids are drawn at the 50% probability level. H atoms are presented as small spheres of arbitrary radius.

**Fig. 2.**
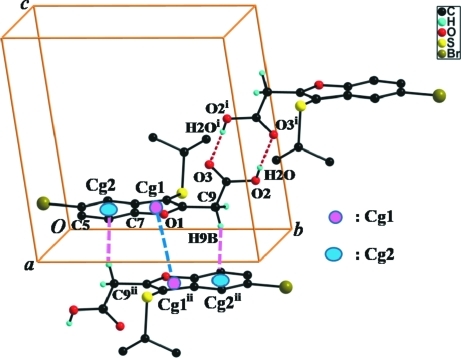
A view of the O—H···O, C—H···π and π–π interactions (dotted lines) in the crystal structure of the title compound. H atoms non-participating in hydrogen-bonding were omitted for clarity. [Symmetry codes: (i) - x + 2, - y + 2, - z + 1; (ii) - x + 1, - y + 1, - z.]

## supplementary crystallographic information

### Comment

Substituted benzofuran derivatives have attracted considerable interest in view of their valuable pharmacological properties such as antibacterial and antifungal, antitumor and antiviral, and antimicrobial activities (Aslam *et al.* , 2009, Galal *et al.* , 2009, Khan *et al.* , 2005). These benzofuran derivatives occur in a wide range of natural products (Akgul & Anil, 2003; Soekamto *et al.* , 2003). As a part of our ongoing study of 2-(5-bromo-1-benzofuran-2-yl) acetic acid analogues (Choi *et al.* , 2009*a*,*b*), we report herein the crystal structure of the title compound.

In the title molecule (Fig. 1), the benzofuran unit is essentially planar, with a mean deviation of 0.007 (1) Å from the least-squares plane defined by the nine constituent atoms. In the crystal structure, the carboxyl groups are involved in intermolecular O—H···O hydrogen bonds (Table 1, first entry  & Fig. 2), which link the molecules into centrosymmetric dimers. These dimers are further packed into stacks along the c axis by an intermolecular C—H···π interaction between a methylene H atom and the benzene ring (Table 1, second entry & Fig. 2), and by a weak slipped π–π interaction between the furan rings of adjacent molecules, with a Cg1···Cg1^ii^ distance of 3.742 (2) Å and an interplanar distance of 3.398 (2) Å resulting in a slippage of 0.713 (2) Å (Fig. 2, Cg1 is the centroid of the C1-C2-C7-O1-C8  furan ring, (ii) -*x*+1, -*y*+1, -*z*.).

### Experimental

Ethyl 2-(5-bromo-3-isopropylsulfanyl-1-benzofuran-2-yl)acetate (428 mg, 1.2 mmol) was added to a solution of potassium hydroxide (337 mg. 6 mmol) in water (10 ml) and methanol (10 ml), and the mixture was refluxed for 5 h, then cooled. Water (10 ml) was added, and the solution was extracted with dichloromethane. The aqueous layer was acidified to pH 1 with concentrated hydrochloric acid and then extracted with chloroform, dried over magnesium sulfate, filtered and concentrated at reduced pressure. The residue was purified by column chromatography (ethyl acetate) to afford the title compound as a colorless solid [yield 86%, m.p. 432–433 K; *R*_f_ = 0.51 (ethyl acetate)]. Single crystals suitable for X-ray diffraction were prepared by slow evaporation of a solution of the title compound in benzene at room temperature.

### Refinement

H atoms in the hydroxy group were found in a different Fourier map and refined freely. The other H atoms were positioned geometrically and refined using a riding model, with C–H = 0.93 Å fo the aryl, 0.98 Å for the methine, 0.97 Å for the methylene, and 0.96 Å for the methyl H atoms. *U*_iso_(H) =1.2*U*_eq_(C) for the aryl, methine, and methylene H atoms, and 1.5*U*_eq_(C) for the methyl H atoms.

### Crystal data

**Table:**

C_13_H_13_BrO_3_S	*Z* = 2
*M**_r_* = 329.20	*F*(000) = 332
Triclinic, *P*1	*D*_x_ = 1.643 Mg m^−^^3^
Hall symbol: -P 1	Mo *K*α radiation, λ = 0.71073 Å
*a* = 7.4689 (3) Å	Cell parameters from 5830 reflections
*b* = 9.9449 (4) Å	θ = 2.3–27.5°
*c* = 10.0653 (4) Å	µ = 3.24 mm^−^^1^
α = 98.415 (2)°	*T* = 296 K
β = 102.146 (2)°	Block, colourless
γ = 110.341 (3)°	0.24 × 0.17 × 0.10 mm
*V* = 665.38 (5) Å^3^	

### Data collection

**Table:**

Bruker SMART APEXII CCD diffractometer	3082 independent reflections
Radiation source: rotating anode	2731 reflections with *I* > 2σ(*I*)
graphite multilayer	*R*_int_ = 0.030
Detector resolution: 10.0 pixels mm^-1^	θ_max_ = 27.6°, θ_min_ = 2.1°
φ and ω scans	*h* = −9→9
Absorption correction: multi-scan (*SADABS*; Bruker, 2009)	*k* = −12→12
*T*_min_ = 0.510, *T*_max_ = 0.738	*l* = −13→13
11869 measured reflections	

### Refinement

**Table:**

Refinement on *F*^2^	Primary atom site location: structure-invariant direct methods
Least-squares matrix: full	Secondary atom site location: difference Fourier map
*R*[*F*^2^ > 2σ(*F*^2^)] = 0.024	Hydrogen site location: difference Fourier map
*wR*(*F*^2^) = 0.064	H atoms treated by a mixture of independent and constrained refinement
*S* = 1.03	*w* = 1/[σ^2^(*F*_o_^2^) + (0.0351*P*)^2^ + 0.1792*P*] where *P* = (*F*_o_^2^ + 2*F*_c_^2^)/3
3082 reflections	(Δ/σ)_max_ = 0.001
169 parameters	Δρ_max_ = 0.52 e Å^−^^3^
0 restraints	Δρ_min_ = −0.30 e Å^−^^3^

### Special details

**Table:**

Geometry. All esds (except the esd in the dihedral angle between two l.s. planes) are estimated using the full covariance matrix. The cell esds are taken into account individually in the estimation of esds in distances, angles and torsion angles; correlations between esds in cell parameters are only used when they are defined by crystal symmetry. An approximate (isotropic) treatment of cell esds is used for estimating esds involving l.s. planes.
Refinement. Refinement of F^2^ against ALL reflections. The weighted R-factor wR and goodness of fit S are based on F^2^, conventional R-factors R are based on F, with F set to zero for negative F^2^. The threshold expression of F^2^ > 2sigma(F^2^) is used only for calculating R-factors(gt) etc. and is not relevant to the choice of reflections for refinement. R-factors based on F^2^ are statistically about twice as large as those based on F, and R- factors based on ALL data will be even larger.

### Fractional atomic coordinates and isotropic or equivalent isotropic displacement parameters (Å^2^)

**Table:**

	*x*	*y*	*z*	*U*_iso_*/*U*_eq_	
Br1	0.19969 (3)	−0.066508 (19)	0.14451 (2)	0.03256 (8)	
S1	0.19347 (6)	0.56388 (5)	0.18733 (4)	0.02002 (10)	
O1	0.71390 (18)	0.55476 (13)	0.17068 (13)	0.0206 (2)	
O2	0.8525 (2)	1.01742 (15)	0.34019 (17)	0.0322 (3)	
H2O	0.925 (4)	1.059 (3)	0.409 (3)	0.050 (9)*	
O3	0.8861 (2)	0.82564 (14)	0.41728 (14)	0.0290 (3)	
C1	0.4055 (2)	0.52528 (18)	0.18780 (17)	0.0180 (3)	
C2	0.4198 (2)	0.38295 (18)	0.17355 (17)	0.0176 (3)	
C3	0.2914 (3)	0.24052 (18)	0.16819 (18)	0.0196 (3)	
H3	0.1627	0.2202	0.1749	0.024*	
C4	0.3645 (3)	0.13091 (19)	0.15246 (18)	0.0213 (3)	
C5	0.5565 (3)	0.1574 (2)	0.14170 (19)	0.0227 (4)	
H5	0.5989	0.0799	0.1316	0.027*	
C6	0.6840 (3)	0.2986 (2)	0.14606 (19)	0.0223 (4)	
H6	0.8119	0.3185	0.1379	0.027*	
C7	0.6116 (2)	0.40805 (18)	0.16307 (17)	0.0190 (3)	
C8	0.5840 (3)	0.62221 (18)	0.18556 (17)	0.0184 (3)	
C9	0.6588 (3)	0.78314 (18)	0.19365 (18)	0.0207 (3)	
H9A	0.5471	0.8131	0.1809	0.025*	
H9B	0.7170	0.8019	0.1172	0.025*	
C10	0.8114 (3)	0.87651 (19)	0.32922 (19)	0.0210 (3)	
C11	0.2061 (3)	0.6058 (2)	0.37390 (19)	0.0252 (4)	
H11	0.0865	0.6237	0.3795	0.030*	
C12	0.3829 (3)	0.7457 (2)	0.4566 (2)	0.0377 (5)	
H12A	0.3768	0.7682	0.5511	0.056*	
H12B	0.3803	0.8259	0.4144	0.056*	
H12C	0.5038	0.7317	0.4565	0.056*	
C13	0.1986 (3)	0.4754 (2)	0.4379 (2)	0.0326 (4)	
H13A	0.3110	0.4516	0.4314	0.049*	
H13B	0.0780	0.3916	0.3882	0.049*	
H13C	0.2020	0.5004	0.5344	0.049*	

### Atomic displacement parameters (Å^2^)

**Table:**

	*U*^11^	*U*^22^	*U*^33^	*U*^12^	*U*^13^	*U*^23^
Br1	0.03694 (13)	0.01654 (10)	0.04563 (14)	0.00761 (8)	0.01796 (10)	0.00961 (8)
S1	0.0193 (2)	0.0200 (2)	0.0201 (2)	0.00800 (17)	0.00334 (17)	0.00490 (16)
O1	0.0187 (6)	0.0170 (6)	0.0213 (6)	0.0032 (5)	0.0045 (5)	0.0012 (5)
O2	0.0399 (8)	0.0150 (6)	0.0274 (8)	0.0067 (6)	−0.0091 (7)	−0.0003 (5)
O3	0.0344 (7)	0.0161 (6)	0.0251 (7)	0.0052 (6)	−0.0054 (6)	0.0028 (5)
C1	0.0203 (8)	0.0175 (8)	0.0139 (8)	0.0061 (7)	0.0033 (6)	0.0025 (6)
C2	0.0196 (8)	0.0176 (8)	0.0125 (8)	0.0058 (7)	0.0024 (6)	0.0012 (6)
C3	0.0208 (8)	0.0190 (8)	0.0179 (8)	0.0061 (7)	0.0063 (7)	0.0041 (6)
C4	0.0249 (9)	0.0168 (8)	0.0186 (9)	0.0049 (7)	0.0051 (7)	0.0035 (6)
C5	0.0270 (9)	0.0209 (8)	0.0203 (9)	0.0119 (7)	0.0041 (7)	0.0025 (7)
C6	0.0190 (8)	0.0246 (9)	0.0205 (9)	0.0077 (7)	0.0043 (7)	0.0011 (7)
C7	0.0193 (8)	0.0189 (8)	0.0137 (8)	0.0034 (7)	0.0028 (6)	0.0013 (6)
C8	0.0214 (8)	0.0177 (8)	0.0134 (8)	0.0065 (7)	0.0027 (7)	0.0015 (6)
C9	0.0220 (8)	0.0160 (8)	0.0183 (9)	0.0026 (7)	0.0029 (7)	0.0032 (6)
C10	0.0215 (8)	0.0161 (8)	0.0217 (9)	0.0041 (7)	0.0051 (7)	0.0027 (6)
C11	0.0270 (9)	0.0309 (10)	0.0212 (9)	0.0140 (8)	0.0094 (8)	0.0056 (7)
C12	0.0431 (12)	0.0360 (12)	0.0241 (11)	0.0091 (10)	0.0077 (9)	−0.0030 (8)
C13	0.0361 (11)	0.0423 (12)	0.0268 (10)	0.0185 (10)	0.0136 (9)	0.0148 (9)

### Geometric parameters (Å, °)

**Table:**

Br1—C4	1.8996 (17)	C5—H5	0.9300
S1—C1	1.7533 (17)	C6—C7	1.378 (2)
S1—C11	1.8380 (18)	C6—H6	0.9300
O1—C7	1.373 (2)	C8—C9	1.484 (2)
O1—C8	1.378 (2)	C9—C10	1.504 (2)
O2—C10	1.308 (2)	C9—H9A	0.9700
O2—H2O	0.74 (3)	C9—H9B	0.9700
O3—C10	1.213 (2)	C11—C12	1.515 (3)
C1—C8	1.354 (2)	C11—C13	1.519 (3)
C1—C2	1.445 (2)	C11—H11	0.9800
C2—C3	1.393 (2)	C12—H12A	0.9600
C2—C7	1.396 (2)	C12—H12B	0.9600
C3—C4	1.382 (2)	C12—H12C	0.9600
C3—H3	0.9300	C13—H13A	0.9600
C4—C5	1.396 (3)	C13—H13B	0.9600
C5—C6	1.383 (3)	C13—H13C	0.9600
			
C1—S1—C11	103.26 (8)	C8—C9—C10	114.05 (14)
C7—O1—C8	105.80 (12)	C8—C9—H9A	108.7
C10—O2—H2O	108 (2)	C10—C9—H9A	108.7
C8—C1—C2	106.08 (14)	C8—C9—H9B	108.7
C8—C1—S1	125.91 (13)	C10—C9—H9B	108.7
C2—C1—S1	127.62 (13)	H9A—C9—H9B	107.6
C3—C2—C7	119.53 (15)	O3—C10—O2	124.59 (17)
C3—C2—C1	134.93 (15)	O3—C10—C9	123.37 (16)
C7—C2—C1	105.53 (14)	O2—C10—C9	112.04 (15)
C4—C3—C2	116.75 (15)	C12—C11—C13	112.01 (17)
C4—C3—H3	121.6	C12—C11—S1	112.33 (13)
C2—C3—H3	121.6	C13—C11—S1	111.88 (13)
C3—C4—C5	123.14 (16)	C12—C11—H11	106.7
C3—C4—Br1	119.60 (13)	C13—C11—H11	106.7
C5—C4—Br1	117.26 (13)	S1—C11—H11	106.7
C6—C5—C4	120.26 (16)	C11—C12—H12A	109.5
C6—C5—H5	119.9	C11—C12—H12B	109.5
C4—C5—H5	119.9	H12A—C12—H12B	109.5
C7—C6—C5	116.58 (16)	C11—C12—H12C	109.5
C7—C6—H6	121.7	H12A—C12—H12C	109.5
C5—C6—H6	121.7	H12B—C12—H12C	109.5
O1—C7—C6	125.78 (15)	C11—C13—H13A	109.5
O1—C7—C2	110.49 (14)	C11—C13—H13B	109.5
C6—C7—C2	123.73 (16)	H13A—C13—H13B	109.5
C1—C8—O1	112.10 (14)	C11—C13—H13C	109.5
C1—C8—C9	131.51 (16)	H13A—C13—H13C	109.5
O1—C8—C9	116.38 (14)	H13B—C13—H13C	109.5
			
C11—S1—C1—C8	−95.71 (16)	C3—C2—C7—O1	−179.98 (14)
C11—S1—C1—C2	92.46 (16)	C1—C2—C7—O1	0.32 (18)
C8—C1—C2—C3	−179.91 (18)	C3—C2—C7—C6	0.6 (3)
S1—C1—C2—C3	−6.8 (3)	C1—C2—C7—C6	−179.08 (16)
C8—C1—C2—C7	−0.27 (18)	C2—C1—C8—O1	0.13 (19)
S1—C1—C2—C7	172.85 (13)	S1—C1—C8—O1	−173.14 (12)
C7—C2—C3—C4	0.0 (2)	C2—C1—C8—C9	178.91 (17)
C1—C2—C3—C4	179.61 (18)	S1—C1—C8—C9	5.6 (3)
C2—C3—C4—C5	−0.2 (3)	C7—O1—C8—C1	0.06 (18)
C2—C3—C4—Br1	179.88 (12)	C7—O1—C8—C9	−178.91 (14)
C3—C4—C5—C6	−0.2 (3)	C1—C8—C9—C10	109.0 (2)
Br1—C4—C5—C6	179.71 (13)	O1—C8—C9—C10	−72.23 (19)
C4—C5—C6—C7	0.8 (3)	C8—C9—C10—O3	8.9 (3)
C8—O1—C7—C6	179.14 (17)	C8—C9—C10—O2	−170.96 (15)
C8—O1—C7—C2	−0.24 (17)	C1—S1—C11—C12	66.06 (16)
C5—C6—C7—O1	179.67 (16)	C1—S1—C11—C13	−60.93 (15)
C5—C6—C7—C2	−1.0 (3)		

### Hydrogen-bond geometry (Å, °)

**Table:**

Cg2 is the centroid of the C2–C7 ring.

**Table:**

*D*—H···*A*	*D*—H	H···*A*	*D*···*A*	*D*—H···*A*
O2—H2O···O3^i^	0.74 (3)	1.90 (3)	2.640 (2)	177 (3)
C9—H9B···Cg2^ii^	0.97	2.72 (1)	3.376 (2)	126.

Symmetry codes: (i) −*x*+2, −*y*+2, −*z*+1; (ii) −*x*+1, −*y*+1, −*z*.
